# Smoking and Rheumatoid Arthritis

**DOI:** 10.3390/ijms151222279

**Published:** 2014-12-03

**Authors:** Kathleen Chang, So Min Yang, Seong Heon Kim, Kyoung Hee Han, Se Jin Park, Jae Il Shin

**Affiliations:** 1 Central Clinical School, Faculty of Medicine, Nursing and Health Sciences, Monash University, Melbourne 3800, Australia; E-Mail: kcha105@student.monash.edu; 2 Department of Pediatrics, Severance Children’s Hospital, Yonsei University College of Medicine, Seoul 120-752, Korea; E-Mail: YANG_SO@yuhs.ac; 3 Department of Pediatrics, Pusan National University Children’s Hospital, Yangsan 626-770, Korea; E-Mail: pedksh@gmail.com; 4 Department of Pediatrics, Jeju National University School of Medicine, Jeju 690-767, Korea; E-Mail: hansyang78@gmail.com; 5 Department of Pediatrics, Ajou University School of Medicine, Daewoo General Hospital, Geoje 656-711, Korea; E-Mail: fli018@hanmail.net

**Keywords:** rheumatoid arthritis, smoking, cyclic citrullinated peptide, synovial fibroblasts, drug response

## Abstract

Rheumatoid arthritis (RA) is a chronic inflammatory disease caused by both genetic and environmental factors. Smoking has been implicated as one of the most important extrinsic risk factors for its development and severity. Recent developments have shed light on the pathophysiology of RA in smokers, including oxidative stress, inflammation, autoantibody formation and epigenetic changes. The association of smoking and the development of RA have been demonstrated through epidemiologic studies, as well as through *in vivo* and animal models of RA. With increased use of biological agents in addition to standard disease-modifying antirheumatic drugs (DMARDs), there has been interest in how smoking affects drug response in RA treatment. Recent evidence suggests the response and drug survival in people treated with anti-tumour necrosis factor (anti-TNF) therapy is poorer in heavy smokers, and possible immunological mechanisms for this effect are presented in the current paper.

## 1. Introduction

Rheumatoid arthritis (RA) is a systemic inflammatory disease characterized by persistent synovitis and the production of auto-antibodies against various factors, including rheumatoid factor (RF) and cyclic citrullinated peptide (CCP) [1,2]. Typically, RA manifests as sustained inflammation of the synovium, which leads to destruction of joints [1,2]. Uncontrolled RA may cause permanent joint damage, reduced mobility and decreased quality of life, as well as cardiovascular and other extra-articular complications [1,2]. It is well established that genetic factors, such as human leukocyte antigen (HLA), and environmental factors, such as infection, ultraviolet, radiation and smoking, can affect the development of various autoimmune diseases [1,2,3]. Among these factors, cigarette smoking significantly increases the risk of not only various types of cancer, cardiopulmonary diseases and infections, but also autoimmune diseases, such as systemic lupus erythematosus and RA [4,5,6,7,8]. Although the exact pathogenic effect of smoking on RA still remains uncertain, several mechanisms have been proposed to better understand how cigarette smoking plays a role in various autoimmune diseases [3,4,5,6,7,8], and citrullination has been reported to be an important factor for the development of RA in the anti-citrullinated protein antibody (ACPA)-positive subset.

In this review, we describe the known immunobiologic effects of cigarette smoking, the epidemiologic studies implicating smoking with increased risk of RA, the effect of smoking on synovial fibroblasts and the impact of smoking on the response to anti-tumour necrosis factor (anti-TNF) therapy.

## 2. Smoking and Risk of RA Susceptibility

Previous epidemiological studies have identified smoking as an important risk factor for RA [9,10,11,12,13,14,15,16,17]. Important studies are summarized in Table 1. Some studies demonstrate that smoking increases the risk of developing RA in men more than in women [10,11,18], while several other reports show that smoking increases the risk of developing RA in women [9,12,16]. Recently, Sugiyama *et al.* [16] conducted the first meta-analysis investigating the significance of smoking as a risk for developing RA, which suggested that smoking is indeed a risk factor for RA in RF-positive men and heavy smokers. The risk of developing RA was approximately twice as high for smokers than for non-smokers. For female smokers, the risk was approximately 1.3-times higher than for non-smokers [16]. Even though many previous studies could not confirm a significant association between smoking and the development RA in women [10,18], Sugiyama *et al.* provided quantitative evidence that smoking is an important risk factor for women in developing RA [16].

Several previous epidemiological studies showed an increasing risk of developing RA with a heavier lifetime burden of smoking [12,19], while a recent report suggested that even light smoking had a connection with the development of RA [17]. Di Giuseppe *et al.* [17] conducted a meta-analysis to quantitatively summarize accumulated evidence regarding the association of lifelong exposure to smoking and concluded that lifelong smoking was positively associated with the risk of RA, even among smokers with overall low lifelong exposure (<10 pack-years). The risk did not further increase with an exposure higher than 20 pack-years in the same study [17].

**Table 1:** Results of studies on smoking and rheumatoid arthritis (RA). SE, shared epitope.

Results	Study Design	Author (Year)
RA was strongly related to smoking in women.	Cohort	Vessey (1987) [9]
Exposure to tobacco smoke or some factor or cluster of factors associated with smoking may trigger the production of rheumatoid factors and, subsequently, contribute to the development of clinically manifest RA in males.	Cohort	Heliövaara (1993) [10]
Current smoking in men was identified as an independent risk factor for RA.	Case-control	Uhlig (1999) [11]
Duration, but not intensity, of cigarette smoking is associated with a modest increased risk of RA in women.	Cohort	Karlson (1999) [12]
Abstinence from smoking may reduce the risk of RA among postmenopausal women.	Cohort	Criswell (2002) [13]
The disease risk of RF-positive RA associated with the SE of HLA-DR is strongly influenced by the presence of an environmental factor (smoking) in the population at risk.	Case-control	Padyukov (2004) [14]
Past and current smoking were related to the development of RA, in particular seropositive RA. Both smoking intensity and duration were directly related to risk, with prolonged increased risk after cessation.	Cohort	Costenbader (2006) [15]
Smoking is a risk factor for RA, especially RF-positive RA in men and heavy smokers. For women, the risk for smokers is about 1.3-times greater than for non-smokers.	Meta-analysis	Sugiyama (2010) [16]
Lifelong cigarette smoking was positively associated with the risk of RA, even among smokers with a low lifelong exposure.	Meta-analysis	Di Giuseppe (2014) [17]

RA: rheumatoid arthritis; RF: rheumatoid factor.

## 3. The Effect of Smoking on the Immune System

### 3.1. Oxidative Stress

Smoking can increase the oxidative stress in the body. Pryor and Stone [20] reported that there are two phases of cigarette smoke: as a particulate (tar) phase and a gaseous (vapour) phase, both of which contain very high concentrations of free radicals. Cigarette smoke is also known to activate endogenous sources of free radicals [20]. It has been reported that oxidative stress increases in rheumatoid inflammation due to impaired antioxidant systems caused by free radicals, which have a role in the etiology of RA [21].

Although the effect of nicotine on RA has been poorly studied, oxidative stress may be triggered by nicotine exposure, causing mitochondrial membrane permeability [20,21]. Barr *et al.* demonstrated that nicotine induced reactive oxygen species levels in a dose-dependent manner in rat mesencephalic cells and also activated inducible nuclear factor-κB by binding to consensus sequences of DNA in electromobility shift analyses [22]. However, nicotine also has an immunosuppressive role, and this will be discussed later (see Section 3.6*.*).

### 3.2. Apoptosis

Smoking can both increase and decrease apoptosis, depending on the cell types [23,24]. It was reported that smoking increases the levels of Fas (CD95) and CD4 T-cells that make cells more vulnerable to apoptosis, leading to high levels of cellular debris, which may not be adequately cleared in autoimmune diseases [23]. Bijl *et al.* reported that smoking was associated with an increase in the percentage of Fas-expressing CD4+ T and B lymphocytes; however, there were no differences between smokers and non-smokers in *in vitro* Fas-induced apoptosis and the percentages of circulating apoptotic lymphocytes between smokers and non-smokers [24]. Cheng *et al.* demonstrated that nicotine induced the apoptosis of human umbilical vein endothelial cells by the Fas/FasL pathway [23]. However, Imirzalioĝlu *et al.* showed that the mean soluble Fas levels were significantly lower in the saliva of smokers’ than in that of non-smokers’, suggesting smoking might induce anti-apoptotic mechanisms in the oral cavity [25]. In RA, the Fas (CD95)-Fas ligand (CD178) apoptotic system is impaired and exhibits inappropriately low activity, leading to persistent synovial inflammation [26]. Intervention, which induces the Fas-FasL pathway, is shown to protect against arthritis in animal models and to reduce arthritic inflammation in human RA studies [26]. Apoptosis-inducing anti-Fas antibodies effectively treated arthritis in several arthritis models, including collagen-induced arthritis, and several other studies using human RA synovium support the utility of agonist intervention on the Fas apoptotic pathway [26,27,28]. These conflicting results regarding the effects of cigarette smoking on apoptosis may require further studies to elucidate its role in the manifestation of RA.

### 3.3. Inflammation

Cigarette smoking acts on both cellular and humoral aspects of the immune system to cause a systemic proinflammatory state [4,29]. The effects of chronic cigarette smoking on innate and adaptive immune responses appear to trigger various morphological, physiological, biochemical and enzymatic changes that lead to impaired antibacterial defences, cellular regulatory activity and inflammatory responses [4,5,29]. In the lungs, alveolar macrophages and other monocytes of the innate system increase significantly in number, which, in turn, increase levels of lysosomal enzymes and secrete elastase responsible for parenchymal and connective tissue damage [4,30]. Elastase might cause such connective tissue damage and lung parenchymal cells, which could contribute to the pathogenesis of chronic obstructive pulmonary disease [30,31]. Bracke *et al.* reported that cigarette smoking increased the expression of matrix metalloproteinase (MMP)-12 (macrophage elastase), which is produced by both macrophages and dendritic cells in the lungs of mice [31]. MMP-12 has also been implicated in the pathogenesis of RA [32,33]. Liu *et al.* reported that RA synovial tissue contained higher levels of MMP-12 messenger RNA compared to osteoarthritis synovial tissue [32], and Wang *et al.* demonstrated that overexpression of MMP-12 in transgenic rabbits significantly enhanced arthritic lesions, resulting in severe synovial thickening, pannus formation, prominent macrophage infiltration at an early stage and a marked destruction of articular cartilage at a later stage [33]. Furthermore, smokers show higher levels of MMPs, particularly proMMP-2 and proMMP-9 [34], and it has been reported that MMP-9 derived from RA synovial fibroblasts may directly contribute to joint destruction in RA [35].

Natural killer (NK) cell activity against cultured melanomas and cancer cells was reported to be reduced significantly in smokers, as well as in animal models [36,37]. However, the role of NK cells in the pathogenesis of RA has not been fully elucidated, and recent reports suggest both protective and detrimental roles of NK cells [37].

Leukocytosis with decreased leukocyte function is commonly found in chronic smoke exposure [4,38,39], and long-term smoking decreases serum immunoglobulin and specific antibody response levels [4,39]. Despite these findings, autoantibody levels (especially anti-nuclear and anti-RF) are higher, which may explain the increased susceptibility to the development of autoimmune diseases, such as RA [1,2,4,5,6,7,8].

In addition, the induction of inflammatory response is evidenced by higher levels of fibrinogen, C-reactive protein (CRP), intercellular adhesion molecule-1 (ICAM-1), E-selectin and higher levels of pro-inflammatory cytokines (e.g., TNF-α, interleukin (IL)-1α, IL-1β, IL-5, IL-6, IL-8, IL-13) in smokers, which is correlated with current and past smoking exposure [4,5,40,41,42,43,44]. Among these, TNF-α, IL-1 and IL-6 have been regarded as particularly important in the pathogenesis of RA, and drugs targeting these cytokines are currently used as an important biologic agent for the treatment of RA [45].

Adhesion molecules have also been studied as a factor in the pathogenesis of RA [43,44]. Klimiuk *et al.* reported that patients with early RA showed high serum concentrations of soluble ICAM-1 and E-selectin levels [43], and the same group also showed that the serum concentrations of soluble ICAM-1 levels correlated with markers of disease activity, such as the erythrocyte sedimentation rate (ESR) and CRP levels [44].

The proinflammatory cytokine, IL-17, which is primarily produced by T helper 17 (Th17) cells, has recently been shown to be an important contributor to RA pathogenesis and chronicity [46]. Smoking is known to increase levels of Th17 and IL-17 [47].

### 3.4. Autoantibodies

Citrullination is the conversion of the amino acid, arginine, in a protein into the amino acid, citrulline, which is not one of the 20 standard amino acids encoded by DNA in the genetic code and is a post-translational modification [48]. Autoantibodies against citrullinated proteins can trigger autoimmune diseases and are sometimes found in high levels in RA [1,2,48]. Smoking is known to trigger HLA-DR-restricted immune reactions to autoantigens modified by citrullination [49]. There is a clear risk of developing anti-CCP antibodies in cigarette smokers, and this risk appears to be linked to disease severity in genetically susceptible individuals with the shared epitope *HLA-DRB1* gene [14,50,51].

### 3.5. Epigenetic Changes

Epigenetic changes, such as DNA methylation, are being explored, as they appear to play crucial roles in gene regulation and development of RA [52]. The methylation status of two clusters in major histocompatibility complex (MHC) regions, as well as individual CpGs, shows that DNA methylation is a potential mediator of genetic risk in susceptible individuals [52]. Recently, it was reported that smoking could lead to extensive genome-wide changes in DNA methylation [53].

### 3.6. Effect of Nicotine on Inflammation

Although increased oxidative stress can be one of the important pathways for RA development [20], nicotine has been reported to paradoxically reduce inflammation in experimental models of RA [54]. Nicotine contributes to immunosuppression, the loss of antibody response and T-cell proliferation in animal models [33,54]. Studies have demonstrated that T-cells from nicotine-treated animals bind to an antigen and then cease transmission of antigen-receptor-mediated signals that trigger the cell cycle and proliferation [54,55]. Moreover, nicotine inhibits TNF-α-induced IL-6 and IL-8 secretion in fibroblast-like synoviocytes from patients with rheumatoid arthritis [56]. These results strengthen the important immunosuppressive function of nicotine in cigarette smoke. However, Yu *et al.* showed the paradoxical effect of nicotine on RA, as nicotine pretreatment aggravated the rat adjuvant-induced arthritis (AIA) model of human RA, while nicotine post-treatment suppressed the disease [57]. Furthermore, the altered severity of AIA directly correlated with the levels of anti-CCP antibodies, Th1/Th17 cytokines and corresponding dendritic cell-derived cytokines [57]. In human studies, Vesperini *et al.* reported that smoking status had no significant effect on RA disease activity and disability, yet did reduce one-year radiographic disease progression in patients with early arthritis in a large prospective cohort; additionally, the study speculated that the anti-inflammatory role of nicotine might explain the lower systemic inflammation and structural disease progression in current smokers with early RA [58]. In a Swedish epidemiologic study, Jiang *et al.* reported that increased risk of RA associated with smoking is most probably not due to nicotine, given that the use of moist snuff (smokeless tobacco containing nicotine) was not associated with the risk of ACPA-positive or ACPA-negative RA [59].

### 3.7. Association of Smoking and Genetic Factors

Both genetic and environmental factors contribute to the development of RA [60]. The genetic contribution to RA pathogenesis has been considered to be as high as 60%, and the HLA region has consistently been shown to have the strongest genetic association with RA [60], especially the *HLA-DRB1* gene, which accounts for two-thirds of the genetic risk of RA. The *HLA-DRB1* shared epitope (SE) encompasses alleles with a similar amino acid sequence at the P4 pocket of the peptide binding region [50]. As shown in a Swedish population case control study, there is a gene-environment interaction between smoking and the *HLA-DRB1* SE genotype [14]. The relative risk of seropositive RA was remarkably high in smokers carrying single SE alleles or double SE alleles; however, these risk factors have not been identified for seronegative RA [14,15]. Similarly, in a Korean population study by Bang *et al.*, the combination of SE alleles and smoking is associated with RA susceptibility regardless of anti-CCP antibody or RF status, although this combination shows stronger effects in anti-CCP and RF-positive RA patients than in anti-CCP and RF-negative RA patients [61]. A recent report by Wagner *et al.* suggested that smoking and possession of *HLA-DRB1* SE alleles contribute to the development of ACPAs in anti-CCP negative RA [62]. In summary, it is well known that there is a gene-environment interaction between smoking and the *HLA-DRB1* SE genotype in seropositive RA; however, further studies are needed to determine such interactions in seronegative RA.

Though RA is significantly associated with the polymorphism of the protein tyrosine phosphatase non-receptor 22 (PTPN22) gene [63], a link between PTPN22 and smoking is yet to be established. A recent meta-analysis by Taylor *et al.* indicates that both smoking and the PTPN22 risk allele are associated with the risk of ACPA positivity [64]. In addition, some gene-smoking interactions, such as glutathione *S*-transferase, *N*-acetyltransferase 2 and the mannose binding lectin genes, have recently been emerging [65,66,67].

## 4. Effect of Smoking on RA Synovial Fibroblasts

Fibroblast-like synoviocytes (FLS) are constituent parts in the thin layers of cells of the synovial membrane and secrete unique proteins, such as lubricin, a protein for joint lubrication in normal tissues [68,69,70]. Synovial hyperplasia is a typical histological feature of RA. Fibroblast-like synoviocytes in RA patients secrete a number of cytokines, chemokines and matrix-degrading enzymes, including IL-1α, IL-1β, IL-6, IL-8 and MMPs, which may create an inflammatory environment in the synovium and contribute to progressive joint destruction [68,69,70].

Cigarette smoke condensate (CSC) induces proinflammatory cytokines, including IL-1α, IL-1β, IL-6 and IL-8, at both mRNA and protein levels in RA-affected FLS [71,72]. Moreover, TNF-α is known to induce the expression of IL-1α, IL-1β, IL-6 and IL-8 mRNA, which are augmented by CSC [71]. Among these proinflammatory cytokines, IL-1 and TNF-α are strongly associated with the pathogenesis in RA [73]. Anti-TNF-α therapies are important in the treatment for RA [73,74] and the significance of the association between current smoking and poor outcome with anti-TNF-α therapies will be discussed in the following section. This relationship can partly be explained by the increase at the mRNA level of IL-1β in RA patient-derived FLS cell lines through the aryl hydrocarbon receptor [72,75].

Sirtuins (SIRTs) influence a wide range of cellular processes, including aging, transcription, apoptosis, inflammation and stress resistance [76]. The TNF-α-induced overexpression of SIRT1 in RA synovial cells contributes to chronic inflammation by promoting IL-6 and IL-8 production and inhibiting apoptosis [77]. On the other hand, CSC also enhances the expression of SIRT6 in FLS from RA patients, which restricts MMP1 production [78]. SIRT6 overactivity can therefore help to reduce the matrix-destructive potential of FLS by CSC stimuli.

Citrullinated calreticulin is overabundant in FLS from RA patients, which introduces the possibility of a new mechanism between gene and environment in RA [79]. Heat shock proteins (HSP) are referred to as stress proteins, and their upregulation is recognized as a stress response [80]. Heat shock proteins, including DnaJB4, DnaJC6, HspB8 and Hsp70, are upregulated in the synovial tissues of smokers with RA, in contrast to non-smokers with RA [81]. They have been described to increase the production of inflammatory cytokines and of matrix-destructive molecules via Toll-like receptors stimulating FLS [81].

## 5. Effect of Smoking on Drug Response in RA

Recent studies have shown an influence of cigarette smoking on RA patients’ response to anti-rheumatic drugs [73,74,82,83]. A study by Hyrich *et al.* from the British Society for Rheumatology Biologics Register found that RA patients who smoke show a reduced clinical response to infliximab, an anti TNF-α drug (OR (95% CI) 0.77 (0.60–0.99)) [73]. Following that study, Mattey *et al.* also demonstrated that poor response to the drug is linked to the pack-year history of smoking, as well as the smoking status of patients at the initiation of anti TNF-α drug treatment, especially infliximab [82]. More recently, Abhishek *et al.* found in their multivariate analysis that smokers taking an anti TNF-α drug have a reduced chance of achieving a moderate response on the European League Against Rheumatism (EULAR) response criteria compared to non-smokers. (OR (95% CI) 0.20 (0.05–0.83), *p =* 0.03) [83]. However, there have been no studies on the effect of smoking on the response to tocilizumab or rituximab.

### 5.1. Serology

A number of mechanisms have been suggested with aims to explain the relationship between smoking and the poor response to antirheumatic drugs. One such suggestion attributes poor response to RF-positivity via high levels of titre [84,85]. Another implicates increased IgA RF in the correlation of poor response with TNF-α antagonists and high levels of RF titre [85]. Lastly, some studies have linked poor response to anti-TNF therapy to high frequencies of anti-CCP antibodies along with high RF titre [86,87].

### 5.2. Cytokines

As mentioned above, smoking is associated with high concentrations of inflammatory cytokines [5,40,41,42,43,44,88]. Smokers had higher ratios of TNF-α/soluble TNF receptor (sTNFR) than non-smokers [88]. Both the increased production of TNF-α by T-cells and higher TNF-α/sTNFR ratios were correlated with the intensity and duration of smoking [88]. Glossop *et al.* proposed that higher levels of TNF-α or ratios of the TNF-α/sTNFR in smokers might be associated with TNF-α antagonist treatment resistance [88]. Tollerud *et al.* demonstrated that smokers had substantially higher levels of serum soluble IL-2 receptor (sIL-2R) [89,90]. Moreover, Kuliala *et al.* showed that a low serum sIL-2R level predicts rapid response to infliximab, a TNF-α antagonist [91]. Moreover, Shin *et al.* suggested that sIL-2R can influence the response to infliximab in RA patients [92]. It is possible that IL-2-sIL-2R system activation affects the response to TNF-α antagonist treatment of RA patients who smoke [92].

### 5.3. Pharmacokinetics and Pharmacodynamics

Apart from changes in serology and inflammatory systems, other factors affecting the pharmacological action of anti-rheumatic drugs may contribute to poor responses. Metsios *et al.* found that RA patients who smoke have higher basal metabolic rates than non-smokers [93]. They suggested that the metabolism of anti-rheumatic drugs can be accelerated in a state of high metabolic activity [93]. Furthermore, Westhoff *et al.* proposed that a higher dose of disease-modifying antirheumatic drugs (DMARDs) may be needed for RA patients who smoke, as smoking diminishes the potency of anti-rheumatic drugs [94]. Moreover, a recent study by Stamp *et al.* showed that smoking could lower the levels of methotrexate polyglutamates [95]. Another possibility is that smoking may increase the production of human anti-chimeric antibodies (HACA) against infliximab. Such antibodies lower serum infliximab concentrations, reducing the response to TNF-α antagonist treatment [96,97].

Although the specific causes remain under study, much research has revealed that smoking aggravates RA and decreases response to TNF-α antagonist treatment. In order to elucidate the exact mechanisms of drug resistance of RA patients who smoke, future studies that include variations in pharmacokinetics (e.g., interference with absorption, clearance of drug) are required.

## 6. Effect of Smoking on Extra-Articular Manifestations in RA

Extra-articular manifestations (EAM) of RA include various disease manifestations and the incidence of EAM is about 20%–45% in RA [98,99,100,101,102]. The recognition of EAM may be important, because it is associated with disease activity and greater mortality in RA [98,99]. EAM include rheumatoid nodules, rheumatoid vasculitis, polyneuropathy, pleuritis, interstitial lung disease with fibrosis, pericarditis, haematological abnormalities, some types of ocular inflammation and secondary Sjögren’s syndrome [98,99,100,101,102]. There have been some reports on the relationship between smoking and EAM in RA [100,101,102,103,104].

In a Swedish population, Turesson *et al.* reported that the main predictors of severe EAM were smoking at RA diagnosis (risk ratio = 2.94) and early disability (Steinbrocker Classes III–IV at diagnosis) (risk ratio = 2.45) in a multivariate analysis [100]. In a Korean cohort, Kim *et al.* demonstrated that the development of EAM was closely associated with smoking (odds ratio = 5.260) in addition to a positive anti-CCP antibody (odds ratio = 5.006), alcohol consumption (odds ratio = 0.218) and disease duration (odds ratio = 1.061) in a multivariate logistic regression analysis [101]. In a Brazilian population, Moura *et al.* showed that pulmonary manifestation, rheumatoid nodules and Sjögren’s syndrome were the most common EAM, which were associated with longer disease duration and current smoking habit (*p* < 0.05) [102]. Nyhäll-Wåhlin *et al.* showed that patients with RA who developed severe EAM were more often current smokers and had a higher mean disease activity score, functional disability and CRP at baseline [103]. Because the incidence and kinds of EAM in RA and the cumulative exposure of smoking may be different among study groups, further studies are necessary to elucidate the relationship between each EAM and the cumulative dose of smoking exposure.

## 7. Concluding Remarks and Future Perspectives

This review summarized the possible link between smoking and the susceptibility or drug response of RA by genetic and immunologic mechanisms. The immunological changes that cause RA in smokers have been traced back to several mechanisms, which especially affect genetically predisposed individuals. Persistent inflammation due to oxidative stress, proinflammatory state, autoantibody production and epigenetic effects might be implicated in the autoimmunity of RA. Recent studies also provide evidence that clinical responses to anti-TNF drugs used to treat RA may be adversely affected by smoking, and smoking may be related to EAM in RA. The effect of smoking on the radiographic progression of RA is not well established and controversial [104,105]. De Rooy *et al.* reported that the effect of smoking on joint damage was mediated via ACPA, and smoking was not an independent risk factor for radiological progression in RA in a meta-analysis of six cohorts [104]. However, a recent study showed that smoking was a strong independent risk factor for radiographic progression in early RA [105]. These differences may be related to several factors, such as different quantification of smoking, a measurement period of radiographic damage, gender or the presence of autoantibodies. Due to the association of smoking with accelerated atherosclerosis, increased cardiovascular risk and the development of various types of cancer, in addition to the influence on the response to anti-rheumatic drugs in RA patients, it is essential to inform RA patients regarding these hazardous effects of smoking with RA.

Further studies are necessary to evaluate other pathophysiologic mechanisms of the associations between smoking and RA and to elucidate whether the interaction of smoking with other toxic environmental factors might be more hazardous and increase the risk of developing RA in the future.
